# The Connection Between Spatial and Mathematical Ability Across Development

**DOI:** 10.3389/fpsyg.2018.00755

**Published:** 2018-06-04

**Authors:** Christopher J. Young, Susan C. Levine, Kelly S. Mix

**Affiliations:** ^1^Department of Psychology, University of Chicago, Chicago, IL, United States; ^2^Department of Human Development and Quantitative Methodology, University of Maryland, College Park, MD, United States

**Keywords:** spatial cognition, mathematical concepts, factor analysis, statistical, developmental psychology, process modeling

## Abstract

In this article, we review approaches to modeling a connection between spatial and mathematical thinking across development. We critically evaluate the strengths and weaknesses of factor analyses, meta-analyses, and experimental literatures. We examine those studies that set out to describe the nature and number of spatial and mathematical skills and specific connections between these abilities, especially those that included children as participants. We also find evidence of strong spatial-mathematical connections and transfer from spatial interventions to mathematical understanding. Finally, we map out the kinds of studies that could enhance our understanding of the mechanisms by which spatial and mathematical processing are connected and the principles by which mathematical outcomes could be enhanced through spatial training in educational settings.

## Introduction

Spatial ability contributes to performance in science, technology, engineering, and mathematics (STEM) domains even controlling for verbal and mathematical abilities (Shea et al., 2001; Wai et al., 2009). In addition, spatial reasoning task performance has been found to correlate with mathematical task performance (e.g., Dehaene et al., 1999), suggesting that spatial reasoning skills overlap with, and could be necessary for, mathematical reasoning skills (Tosto et al., 2014). One correlation supported by cognitive and developmental research is between representations of numerical and spatial magnitudes. Spatial skills have been found to correlate with numerical magnitude representations across broad age ranges, from preschoolers (Gunderson et al., 2012) to adults (Sella et al., 2016). Further, spatial and numerical magnitude representations have overlapping neural representations (Piazza et al., 2007; Holloway et al., 2010). In this article, we review evidence for the connections between spatial and mathematical skills across development that has been gleaned from factor analyses, meta-analyses, and experimentation. We then suggest productive ways to elucidate spatial-mathematical connections and discuss ways that modeling could be used to improve mathematics learning.

## Factor analysis

Both spatial and mathematical ability have been investigated since the early days of psychological science using factor analytical methods that sought to map the “structure of the intellect” (Spearman, 1927; Thurstone, 1938). This research showed a connection between spatial and mathematical domains, yet the mechanisms by which training spatial thinking can promote mathematical thinking are still not well understood. Across various factor analyses of spatial skills that have been conducted in adults, the most consistent finding is that there are multiple spatial skills, such as spatial visualization (imagining transformations) and spatial relations and spatial orientation (perceiving object position and angle) (Michael et al., 1957; McGee, 1979; Lohman, 1988; Carroll, 1993). Factor analyses carried out on mathematical measures over various ages have revealed latent factors that do not appear to be specific to mathematics (e.g., deductive reasoning and adaptability to a new task among 10th grade students, Kline, 1960; abstraction, analysis, application among elementary school students Rusch, 1957). These studies are notable in that some theorists have found evidence of a spatial factor in mathematics (e.g., Kline, 1960; Werdelin, 1966) and others have argued that there is a spatial sensorimotor intelligence factor important to mathematical reasoning (Coleman, 1960; Skemp, 1961; Aiken, 1970).

### Separate but correlated spatial and mathematical thinking factors

While many studies have found evidence of connections between spatial and numerical tasks in young children, only recently have studies explored the factor structure of their spatial and mathematical skills. Mix et al. (2016, 2017a) have used factor analyses to examine the connections among a broad range of mathematical and spatial tasks in elementary school age children. Mix et al. (2016) administered a battery of tasks that had the greatest likelihood of showing spatial-mathematical connections based on the literature, including connections between (1) spatial visualization and complex mathematical relations, (2) form perception and symbolic reasoning, and (3) spatial scaling and numerical estimation (Landy and Goldstone, 2010; Slusser et al., 2013,; Thompson et al., 2013, respectively). These tasks were included in order to identify which underlying variables that connect spatial and mathematical domains in kindergarten, third and sixth grades.

Between kindergarten and sixth grade range, all spatial tasks loaded together on a distinctly spatial factor, and all mathematical tasks loaded on a distinctly mathematical factor (Mix et al., 2016, 2017a). However, there was a moderate correlation between the two factors (*r*s = 0.50–0.53), even when controlling for verbal ability, suggesting that although the spatial and mathematical domains are distinct, there is a significant relation between these domains. Even though verbal ability accounted for a significant portion of variance in mathematical skills in each grade tested, spatial skills accounted for a greater proportion of variance (Mix et al., 2016). Cross-loadings between the spatial and mathematical factors and tasks in the two domains also indicate specific connections. In kindergarteners, mental rotation was significantly related to the mathematical factor, whereas in sixth graders visuospatial working memory and form copying were significantly related to the mathematical factor. One possible explanation for the change in cross-loadings over development is that mathematical thinking relies at first on dynamic, object-focused spatial processes (mental rotation) and later on more static, memory-related spatial processes (visuospatial working memory and visuomotor integration).

### Strengths and limitations of factor analysis evidence

Factor analysis is a useful tool for isolating the source of correlations and removing measurement error (Bollen, 1989) as well as for testing competing theories (Gerbing and Hamilton, 1996; Tomarken and Waller, 2005). However, factor analysis requires a large number of participants over a breadth of tasks in a domain to achieve a stable structure (Hair et al., 1995; MacCallum et al., 1999). The biggest limitation of factor analysis lies in the theorist; interpretation of results is a large part of proper factor analysis because the results do not uniquely point to any single interpretation of the meaning of the underlying latent variables that are revealed (Armstrong and Soelberg, 1968; Rummel, 1970). Thus, when relations do emerge from factor analysis, other methods must be used to establish mechanisms underlying these relations.

## Meta-analytic and experimental studies

In addition to factor analyses, researchers have tackled the question of how the domains of space and math are connected through targeted experimental studies and meta-analyses. In this section, we outline prominent theories about the divisions in each domain and evidence for correlations between spatial and mathematical skills. Understanding these theories is important because they can help us to understand which particular facet or type of spatial thinking is linked to a particular type of mathematical thinking.

One comprehensive meta-analysis of spatial skills training by Uttal et al. (2013) assumed a 2 × 2 typology supported by behavioral (Newcombe and Shipley, 2015) and neurological evidence (e.g., Chatterjee, 2008). Specifically, relations between objects are processed differently than relations of feature within an object (the extrinsic-intrinsic division). Further, spatial information conveyed by a static viewing of objects and scenes is processed differently than movements and transformations of these objects and scenes (the static-dynamic division). In their factor analysis testing the 2 × 2 typology, (Mix et al., under review) found evidence for distinct spatial factors for tasks involving within object (intrinsic) vs. between object (extrinsic) information, but did not find support for spatial tasks separating according to the static-dynamic distinction (Mix et al. under review). Echoing this finding, Kozhenikov et al. found evidence that some children process spatial information intrinsic to objects better (object visualizers) whereas others process spatial information that involves between object relationships better (spatial visualizers) but did not find that these groups of children differed in their ability to process dynamic and static imagery (Kozhevnikov et al., 2005).

The number and nature of basic mathematical skills that underlie mathematical thinking are also in question. For example, a distinction has been made between core number systems that represent exact and approximate number (Carey, 2004; Feigenson et al., 2004), between core systems for approximate number and ratio (Matthews and Hubbard, 2017), and between core approximate number system and exact number ability enabled by symbolic knowledge (e.g., Carey, 2004). However, the debate about the systems that characterize mathematical thinking has taken on a more pragmatic turn than those concerning spatial thinking. For instance, there are direct educational implications to whether core mathematical skills facilitate later symbolic mathematical understanding and achievement and how the latter might affect the former (e.g., Feigenson et al., 2013; Schneider et al., 2017) or whether mathematics is better taught through concepts or procedures (e.g., Schoenfeld, 1985), or abstractly or concretely (e.g., Kaminski et al., 2009). Researchers also debate which kinds of early mathematical skills relate to later mathematical achievement (e.g., understanding patterns, Rittle-Johnson et al., 2017; thinking symbolically, Schneider et al., 2017, or one's ordinal vs. absolute sense of number Lyons et al., 2014). These debates raise interesting questions about the connection between spatial skills, early mathematical skills, and later mathematical achievement. For example, does a particular type of spatial skill relate to children's ability to learn particular early mathematical skills more quickly, and are these the early mathematical skills that relate most strongly to later mathematical achievement?

### What skills are used in both spatial and mathematical problems?

Certain connections between specific spatial skills and mathematical skills have been observed (e.g., visuospatial working memory and computation, Raghubar et al., 2010) whereas others have not (e.g., between disembedding shapes from scenes and parsing information in charts, Clark, 1988) with little explanation as to why this is the case (for a review of these connections see Mix and Cheng, 2012). One frequently observed connection is between mental rotation and various math skills, across age and development and with a variety of different mental rotation task characteristics (Table 1). However, little is known about the processes that account for this connection, or whether there are other spatial-mathematical connections that may be even stronger. Thus, this correlational type of evidence fails to provide support for the theory that certain specific spatial skills are particularly important for mathematics achievement nor how they enable better performance and learning of specific mathematical skills. Answers to these questions are of high importance to successfully incorporating spatial learning into mathematical curricula.

**Table 1:** Observed relations between mental rotation and mathematical skills.

**References**	**Average age**	**Mental rotation task characteristics**	**Measure or mathematical skill**
Gunderson et al., 2012	5.4	Children's mental transformation task, 2D figure rotation/construction (Levine et al., 2018)	Number une estimation, appoximate calculation
Kyttälä et al., 2003	6.16	Novell “'Troll” task, 2D, same/different choice	General math skill
Carr et al., 2008	7.5	Cube rotation (Vandenberg and Kuse, 1978)	Arithmetic
Battista, 1990	12	Purdue spatial visualizaiton Test, 3D images rotation (Guay, 1976)	Logical reasoning, geometric knowledge and problem solving
Hegarty and Kozhevnikov, 1999	12.08	Primary mental abilities, 2D rotation/figure completion (Thurstone, 1938)	Problem solving skill
Delgado and Prieto, 2004	13	Cube rotation (Peters, 1995)	Geometry, word problems
Casey et al., 1997	13.8	Cube rotation (Vandenberg and Kuse, 1978)	Geometry, SAT math
Kyttälä and Lehto, 2008	15.5	Cube rotation (Vandenberg and Kuse, 1978)	Mental arithmetic, geometry, word problems
Reuhkala, 2001	15.5	Cube rotation (Vandenberg and Kuse, 1978)	Math skill (mental arithmetic, algebra, geometry)
Geary et al., 2000	19	Cube rotation (Vandenberg and Kuse, 1978)	Arithmetic
Thompson et al., 2013	21.26	Cube rotation (Peters, 1995)	Compatibility effect of number comparison

Moving beyond correlational studies, studies that have measured the impact of training mental rotation on specific mathematical skills, have not yielded consistent findings, with some finding evidence of transfer (e.g., Cheng and Mix, 2014; Lowrie et al., 2017) and some not finding such evidence (Hawes et al., 2015b; Xu and LeFevre, 2016). There is little explanation, and as of yet no meta-analysis, to compare these cross-domain training studies or determine the overall effectiveness of training any individual spatial skills to improve mathematical reasoning. In the next section, we argue that modeling and testing the processes involved in performing specific spatial and mathematical tasks can help us understand the connections between these two domains.

## Cognitive process models

Cognitive process models provide an account of the mental processes engaged when performing a specific task. What cognitive process or processes actually drive performance on a spatial task? Answering this question would also allow us to understand the mechanism that accounts for the connection between spatial skills, like mental rotation, and performance on mathematical tasks such as missing term problems (Cheng and Mix, 2014). This in turn would inform educational efforts to improve spatial thinking in ways that would be most helpful to mathematical thinking.

What is known about the processes used for spatial skills? Various studies have supported substantive divisions between particular kinds of spatial skills, e.g., the intrinsic-extrinsic divide separating tasks such as mental rotation from perspective taking (Huttenlocher and Presson, 1973; Kozhevnikov and Hegarty, 2001). However, studies with kindergarten through sixth grade children also show a great deal of overlap among a wide range of spatial skills (Mix et al., 2016, 2017a). Further, certain spatial skills, notably mental rotation and visuospatial working memory, have been found to cross-load onto a mathematical factor at particular grade levels. An important next step is to examine process models of spatial skills and how they are manifested (or not) on mathematical tasks, as illustrated below regarding mental rotation.

### A process view of mental rotation

Mental rotation was first described based on the finding that time to simulate the rotation of an object was related to the angle through which the object was rotated (Shepard and Metzler, 1971). Cognitive process models, supported by empirical studies, reveal that mental rotation actually involves multiple, non-obvious sub-components. Behavior is best fit by a model that involves carrying out small, successive, variable transformations, rather than a single rotation (Provost and Heathcote, 2015) and empirical work suggests that individuals actually rotate just one part of the object rather than all parts of the whole object (Xu and Franconeri, 2015). Further, modeling shows that the type of mental rotation problem influences the process that is engaged; when rotating complex stimuli, participants tend to be slower (Bethell-Fox and Shepard, 1988; Shepard and Metzler, 1988), which has been fit by computational models of mental rotation where task relevant features of the object are focused on and task irrelevant features are ignored (Lovett and Schultheis, 2014). Participants also frequently err in problems with complex stimuli by selecting the mirror image of the correct choice that is rotated to the same degree as the correct choice (e.g., among children Hawes et al., 2015a,b), a pattern of data that is explained by a model that parameterizes “confusability” between the target and its mirror (e.g., confusing a “d” for a “b,” Kelley et al., 2000). Relatedly participants tend to use a fast flipping transformation akin to matching features for simple, 2D stimuli, which models of mental rotation have taken this into account (Kung and Hamm, 2010; Searle and Hamm, 2012). The varied components described by these models make clear that mental rotation is not a simple process, and that there are many steps needed to succeed at a mental rotation task.

Each of these modeled components of mental rotation performance has a potential role to play in the observed relationship between mental rotation and various mathematical skills over the course of development. If spatial constructs are actually based on wide-ranging processes it opens up the hypothesis space to determine the source of connections between spatial and mathematical thinking. Rather than a simple connection between two monolithic skills, there are numerous possible connections based on the components of each, and possibly even multiple ways a spatial skill can act in a single math problem. The work of figuring out which components are critical to the observed relation between spatial and mathematical skills, while daunting, is needed in order to unpack what otherwise are opaque connections.

**Figure 1:**
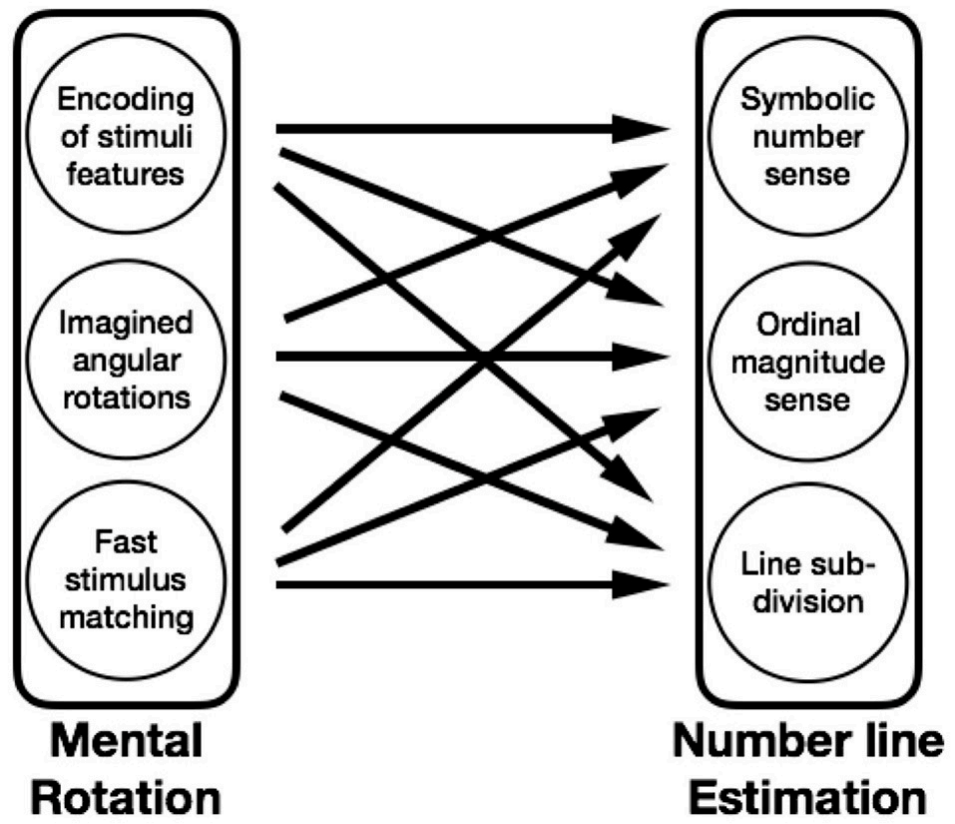
Potential connections among spatial and mathematical skill components.

To take one example, Gunderson et al. (2012) observed a predictive relationship between young children's mental transformation skill and their number line estimation. Individual differences in mental rotation performance could have arisen as a difference in any of the components identified above: the ability to carry out rotations, to focus on relevant spatial information, or to carry out non-rotational stimulus matching. Similarly, the number line estimation task, where participants are asked to determine the position of a number along a labeled line, could be decomposed into several components as well (e.g., accessing a representation of a number's magnitude when cued by its symbol, ordering those magnitudes precisely on a continuous number line, spatially subdividing the line at salient landmarks, Siegler and Opfer, 2003). Any or all of these components might be the source of the connection between number line estimations and spatial skill (see Figure 1). By designing studies that control for and model the components of both spatial and mathematical tasks, it should be possible to identify and understand the mechanisms that explain links between spatial and mathematical thinking. This approach compliments and enriches the work focused on looking at the latent structure of skills, while not dwelling on an explanation of any one task but focusing on explaining important connections between latent skills.

## Educational implications

Meta-analyses provide strong evidence that training spatial skills in the laboratory result in significant improvements and transfer to other spatial skills (Uttal et al., 2013). However, evidence is more mixed about training spatial skills to improve mathematical skills (e.g., Cheng and Mix, 2014; Hawes et al., 2015b; Simons et al., 2016; Lowrie et al., 2017). Broader training regimes in and out of the classroom have helped to improve mathematics performance in multiple age groups (e.g., Witt, 2011; Sorby et al., 2013; Bruce and Hawes, 2015), and more generally, spatial thinking has been shown to be a significant predictor of STEM outcomes, even controlling for mathematical and verbal thinking (Wai et al., 2009).

One finding substantiated by factor analyses and interventions is that spatial skills are more closely related to novel mathematical and scientific content than to STEM skills that are more familiar (Stieff, 2013; Mix et al., 2016), suggesting that it may be particularly important to provide students with spatial scaffolding when students are learning a new mathematical concept. Another set of findings suggests that providing students with a repertoire of spatial tools, such as gesture, rich spatial language, diagrams, and spatial analogies, (Newcombe, 2010; Levine et al., 2018) can facilitate their spatial thinking. Moreover, these tools, as well as 3-D manipulatives (Mix, 2010) have been found to facilitate learning mathematical concepts (e.g., Richland et al., 2012; Verdine et al., 2014; Hawes et al., 2017; Mix et al., 2017b). An overarching principle to guide the use of spatial thinking and tools in education is that supporting spatial thinking and learning beginning early in life may result in improvements in mathematics understanding, based on the general connection between spatial and mathematical factors as well as evidence that training particular spatial skills shows some transfer to mathematics skills. A promising avenue for future work is not just to support spatial thinking in general, but to show students how they can use this kind of thinking to solve particular kinds of mathematical problems (Casey, 2004).

## Conclusions

In this review, we critically evaluate the contributions of the factor analytic method to identifying and elucidating the connection between spatial and mathematical thinking across development. We highlighted a central gap in our knowledge—understanding the mechanisms connecting spatial and mathematical skills—which can be better addressed through targeted experimental studies that are informed by process models than by factor analytic studies. The findings that can emerge from this approach are important for increasing our basic understanding of why spatial and mathematical thinking are connected. They also hold promise for informing educational efforts to increase mathematical achievement by strengthening spatial thinking by training spatial skills, by encouraging the use of spatial tools, and by showing children how they can deploy these skills and tools to solve particular kind of mathematical problems.

## Author contributions

CY wrote the original draft and led efforts to refine subsequent drafts for this article. All authors worked on a related chapter; SL and KM contributed substantially to the writing and editing of this article.

### Conflict of interest statement

The authors declare that the research was conducted in the absence of any commercial or financial relationships that could be construed as a potential conflict of interest.
